# Supplementary material for: Learning curve and surgical outcome of robotic assisted colorectal surgery with ERAS program
Source: Sci Rep. 2022 Nov 29;12:20566. doi: 10.1038/s41598-022-24665-w (PMC9709162; doi:10.1038/s41598-022-24665-w)
Supplement: Supplementary file 2 — Supplementary Information 2. [file 41598_2022_24665_MOESM2_ESM.zip › RAL Raw data-Ñ[▒K/088/OP N_ñw╝╨░O▒Kñσ.pdf]

W95 / W95

臺中榮民總醫院
手術室護理記錄

[redacted]

## 基本資料

- 室別： 開刀房-17室，手術科別： CRS，手術類別： 常規，日期： 2021/06/02
- 進入時間： 0910 ； 麻醉開始時間： 0930 ； Time Out時間： 1040 ； 劃刀時間： 1041 ； 手術結束時間： 1400 送出時間： 1415 ；
- 麻醉方式： GE
  診斷： Malignant tumor of A-colon
  手術名稱： Robot Assisted Colectomy(R)
  手術局部用藥： NIL
  術前預防性抗生素：
  - 抗生素名稱一： Veterin 1GM IVD/由麻醉護理人員給予
  - 時間： 1030
  - 上次簽章: 紀致均

  ，追加預防性抗生素：
- 手術醫師： 林俊余，楊曙榮，張嘉育
- 麻醉醫師： VS 齊光彪
- 
  - 刷手護士A ： 陳盈妤，時間： 0910，刷手護士B ： 許雅芳，時間： 0910，接替刷手一： 歐真勤，時間： 1130，接替刷手二： 許雅芳，時間： 1200
  - 流動護士A ： 紀致均，時間： 0910，接替流動一： 陳盈妤，時間： 1230

## 皮膚狀況

A-V Shunt： 無
術前： 完整
記錄者： 上次簽章: 紀致均
術後： 完整
記錄者： 陳盈妤

## 手術準備

手術臥式 單一手術臥式

- 
  - 手術臥式： 仰臥，NIL
  - 使用附件： 其它： 肩海綿墊、silicon pad
  - 皮膚準備： 10%Aq Betadine，Hibiscrub，2% Chlorhexidine，其它： NIL
  - 電燒： 使用電燒: 電極片位置： 右大腿，雙極電燒，記錄者： 上次簽章: 紀致均

止血帶T： 無

## 植入物

- 植入物名稱/號碼/數量/部位： 達勝/Davinci EndoGIA/45-4.3mm x1,45-3.5mm x2/colon

, 記錄者： 上次簽章: 紀致均

## 標本送檢

- 細菌培養 無
- 細胞學 無
- 冰凍切片 無
- 病理標本
  組織名稱一： R't colon& L.N 211
  ,
  組織名稱二： SMV L.N 213
  ,
  組織名稱三： R't colic L.N 212
  ，更多組織名稱
  - 組織名稱四： IC L.N 212

- 截肢 無

標本交付 標本： 4 袋，標本： 0 盒，標本： 0 瓶，細胞學： 0 管，Culture Tube： 0 管，被交付醫師： CR 楊曙榮，

- 記錄者： 上次簽章: 陳盈妤
- 時間： 1345

## 傷口術後敷料

傷口敷料： 傷口敷料： 無

- 請於圖上標示部位：

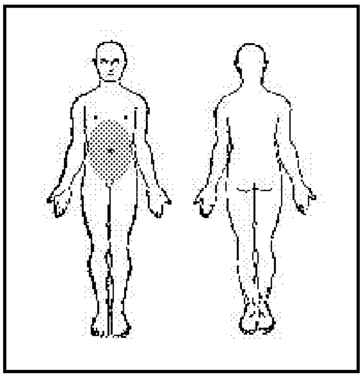

- 傷口敷料： 傷口敷料片

## 導管

| 導管 | 天數 | 導管資訊 | 插入日期 | 插入時間 | 更換日期 | 拔除日期 | 拔除時間 | 拔除原因 | 預計拔除/更換 | 預計拔除/更換日期 |
|---|---|---|---|---|---|---|---|---|---|---|
| A-line (1) | 1 | 號碼:20 G; 部位:右手; | 2021/06/02 | 1020 | | | | | | |
| Double Lumen CVC(1) | 1 | 號碼:8 Fr.; 深度:fix15cm; 部位:jugular (右); 象限:RU; | 2021/06/02 | 1025 | | | | | | |
| Foley(1) | 1 | 號碼:16 Fr.; 部位:膀胱; | 2021/06/02 | 1030 | | | | | | |

## 用物計數

- 手術中出血量： minimal c.c.
- 手術中尿量： 150 c.c.
- 
    - 其它： 無
    - NIL

用物計數： 多切口同時縫合

- 1.器械： 正確，2.縫針： 正確，3.刀片： 正確，4.紗布： 正確，5.腹部墊： 未使用，6.紗球： 未使用，7.PEANUTS ： 未使用，8.紗條： 未使用，9.絲帶： 未使用，10.針頭： 未使用，11.Vessel loop： 未使用，12.Boots： 未使用，13.Jelco： 未使用，14.棉條： 未使用，
    - 第一次：
        - 刷手護士 ： 許雅芳
        - 流動護士： 上次簽章: 陳盈妤
        - 時間： 1325

    ,
    - 第二次：
        - 刷手護士 ： 許雅芳
        - 流動護士： 上次簽章: 陳盈妤
        - 時間： 1330

    ,
    - 第三次：
        - 刷手護士 ： 許雅芳
        - 流動護士： 上次簽章: 陳盈妤
        - 時間： 1340

## 備註

備註： Xi Davinci Scope No.1

- 記錄者： 上次簽章: 紀致均
- 時間： 0930

填寫護理人員 陳盈妤

正式摘要

W95 / W95

doctorRank:B
